# Angehörige von Patienten mit Demenz als Bindeglied und Vermittler im Akutkrankenhaus

**DOI:** 10.1007/s00391-021-01892-w

**Published:** 2021-04-20

**Authors:** Jasmin M. Greskötter

**Affiliations:** Department für Pflegewissenschaft, Universität Witten/Herdecke, Witten, Deutschland

**Keywords:** Demenzsensibilität, Pflegende Angehörige, Akutstationäres Setting, Begleitung, Fürsprecher, Dementia sensivity, Family member, Hospitalization, Accompany, Advocacy

## Abstract

**Hintergrund:**

Den Bedürfnissen von Patienten mit Demenz kann im organisations- und ablauforientierten Akutkrankenhaus kaum Rechnung getragen werden. Um dennoch eine gelingende Interaktion zwischen Patienten und medizinisch-pflegerischem Fachpersonal herzustellen, wird die Bedeutung von Angehörigen als Bindeglied und Vermittler betrachtet.

**Ziel:**

Aus Publikationen zu ähnlichen Themen wird abgeleitet, welche Bedingungen Angehörige benötigen, um als Bindeglied und Vermittler agieren zu können.

**Material und Methode:**

In den Datenbanken *Medline* (*PubMed*), *Cochrane, CINAHL* und *GeroLit* erfolgte eine systematische Recherche.

**Ergebnisse:**

Aus der vorliegenden Literatur lassen sich Gründe für die Begleitung durch Angehörige skizzieren, ihre Tätigkeiten im Akutkrankenhaus beschreiben sowie hinderliche und förderliche Faktoren darlegen.

**Diskussion:**

Angehörige sind bereits als Bindeglied und Vermittler zwischen den Patienten mit Demenz und dem Krankenhauspersonal tätig. Dabei treten sie für den Patienten als Fürsprecher ein und stehen den Fachkräften als Experten und Berater zur Verfügung. Für das Gelingen dieser Aufgabe gibt es Bedingungen, welche die Vermittlungstätigkeit fördern oder erschweren.

## Abstract (en)

**Background:**

The needs of patients with dementia can hardly be met in an organization- and process-oriented acute care hospital. In order to create a successful interaction between patients and hospital staff, the importance of relatives as a link and mediator is considered.

**Objective:**

From publications on similar topics, it is deduced which conditions relatives need in order to be able to act as a link and mediator.

**Material and methods:**

A systematic search was conducted in the databases of Medline (PubMed), Cochrane, CINAHL, and GeroLit.

**Results:**

From the available literature, the reasons for the accompaniment by relatives can be outlined, their activities in the acute care hospital can be described and hindering and supporting factors can be presented.

**Conclusion:**

Relatives already act as a link and mediator between patients with dementia and hospital staff. In doing so they act as advocates for the patient and are available to the professionals as experts and advisors. To accomplish this task, there are conditions that support or hinder the mediation activity.

Bei ca. 40 % der über 65-jährigen Krankenhauspatienten liegen kognitive Beeinträchtigungen, Demenz oder Delir vor [4, 8, 15, 32]. Diese Neben- oder Begleiterkrankungen beeinflussen die Gestaltung und den Verlauf von Krankenhausaufnahme, -behandlung und -entlassung maßgeblich. Vor allem Patienten mit Demenz erleben Auswirkungen wie zunehmende Verwirrtheit, Angstzustände, Verschlechterung des kognitiven Status, Abbau alltagspraktischer Fähigkeiten, Sedierung, Fixierung und ggf. einen Umzug in eine vollstationäre Versorgungseinrichtung [6, 9–11, 31].

## Hintergrund

Um sich der besonderen Situation von Menschen mit Demenz im Akutkrankenhaus zu widmen, wurden in den letzten Jahren Modellprojekte in diversen Akutkrankenhäusern durchgeführt. National und international ist bekannt, dass folgende organisations- und systembedingte Gegebenheiten für diese Patientengruppe nachteilig sind [9, 17, 29]: eine laute und stressige Umgebung, starre organisatorische Abläufe, Zeitvorgaben, häufig wechselndes Personal, fehlende Möglichkeiten zu Tagesstrukturierung und Beschäftigung. Diese stehen im deutlichen Widerspruch zu den Grundbedürfnissen demenziell erkrankter Personen wie Vertrautheit, Sicherheit, Kontinuität, Beschäftigung und eine ruhige Umgebung [7].

Trotz der zunehmenden Kenntnisse über die Bedeutsamkeit der Bedürfnisse von Personen mit Demenz ist es bislang schwierig, demenzsensible Strukturen im Akutkrankenhaus umzusetzen. Ursächlich sind laut Kirchen-Peters und Krupp [17] das Unterschätzen der Auswirkungen der Demenz auf die Behandlung und medizinisch-pflegerische Versorgung, fehlende Kenntnisse des ärztlichen und pflegerischen Personals im Umgang mit demenziell erkrankten Personen, das Vergütungssystem sowie eingefahrene Organisations- und Kommunikationsstrukturen [17].

Folglich treffen zwei Welten aufeinander: die des vulnerablen Patienten mit einer demenziellen Erkrankung und jene der ablauforientierten Organisation Akutkrankenhaus [9, 17, 23, 28, 30]. Für demenzkranke Patienten bedeutet ein Krankenhausaufenthalt eine extreme Ausnahmesituation. Zusätzlich zur krankheitsbedingten Entfremdung des eigenen Körpers kommt eine fremde Umgebung mit ihren spezifischen Eigenarten, ganz eigenen Regeln und eigener Sprache hinzu [28]. Für das Klinikpersonal ist das Akutkrankenhaus hingegen Alltagswelt, inklusive der damit verbundenen Normalität und Routine [27]. Die Behandlung und Begleitung von Patienten mit Demenz stellen allerdings besondere Anforderungen und bilden einen Sonderfall, dessen Bewältigung das Abweichen von Routine und Normalität erfordert.

Die Interaktion zwischen Patienten und Personal ist aufgrund der Demenz und der damit einhergehenden Abnahme der kognitiven und kommunikativen Fähigkeiten einerseits und der fehlenden Kenntnisse des Personals im Umgang mit Demenzkranken andererseits deutlich erschwert [1, 13]. Daraus resultiert ein Interaktionsdefizit. Dieses steht einer adäquaten Bewältigung des Krankenhausaufenthaltes und einer optimalen Versorgung des Patienten mit Demenz entgegen.

Diese Situation führte zu der Überlegung, was oder wer das Interaktionsdefizit zwischen Patienten mit Demenz und dem Personal im Akutkrankenhaus beeinflussen kann. In den Fokus traten hierbei die Angehörigen der Patienten: Ehe- und Lebenspartner, Geschwister, Kinder, Schwiegerkinder und Enkel [6, 10, 14–16, 21]. In einem weiter gefassten Verständnis gehören auch Freunde und Nachbarn dazu [19].

**Figure 1:**
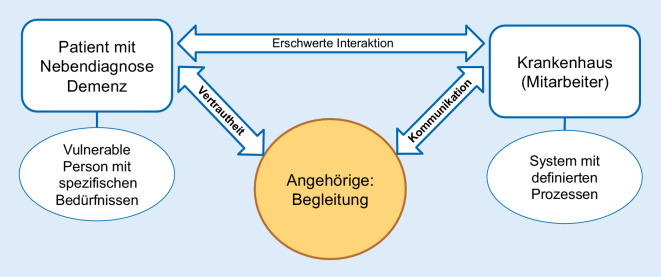

Angehörige sind in der Lage, beide Welten zu erfassen und mit ihnen zu interagieren: Als primäre Bezugsperson für den demenziell erkrankten Menschen kennen sie die Lebensgeschichte, Bedürfnisse, Vorlieben, Ängste und Zugangswege – auch bei eingeschränkter Kommunikationsfähigkeit. Darüber hinaus sind Angehörige in der Lage, die Anforderungen und Funktionsweisen eines Akutkrankenhauses zu verstehen [3, 14–16, 21, 29]. Demnach könnten Angehörige als Bindeglied und Vermittler zwischen den Patienten mit Demenz und den Krankenhausmitarbeitern fungieren und so positiv auf den Interaktionsbereich zwischen Patienten und Personal wirken (Abb. 1).

## Ziel und Fragestellung

Mit der Perspektive von Angehörigen demenziell Erkrankter im Akutkrankenhaus befassten sich Publikationen bislang aus anderen Blickwinkeln (Tab. 1). In diesem Artikel wird der Blick auf Angehörige als Ressource, Bindeglied und Vermittler gerichtet – mit dem Ziel der Beschreibung, welche Bedingungen Angehörige antreffen und benötigen, um diese Rolle wahrnehmen zu können. Die leitende Frage für die Analyse lautete: Wie erleben und beschreiben Angehörige ihre Begleitung eines Patienten mit Demenz im Akutkrankenhaus?

**Table 1:** 

Autor (Jahr)	Jahr	Land	Design	Ziel
Beardon et al. [3]	2018	England	Systematisches Review	Sicht der Angehörigen auf die Qualität der Versorgung von Menschen mit Demenz im Akutkrankenhaus
Boltz et al. [6]	2015	USA	Deskriptive Sekundäranalyse mit einem Mixed-Methods-Ansatz	Faktoren, die zu Angst bei Angehörigen von hospitalisierten Personen mit Demenz beitragen, sowie Perspektiven der Angehörigen auf die akute Krankheit und den Krankenhausaufenthalt
De Vries et al. [10]	2019	Neuseeland	Mixed-Methods-Studie	Erfahrungen der Familienmitglieder von Menschen mit Demenz bei der Unterstützung während der Krankenhausaufnahme
Hynninen et al. [14]	2015	Finnland	Qualitatives, deskriptives Design	Die Behandlung älterer Menschen mit Demenz auf einer chirurgischen Station aus der Perspektive der Patienten und ihrer nahen Angehörigen
Jurgens et al. [15]	2012	England	Qualitative Studie	Erklärung der Unzufriedenheit von Angehörigen mit der Versorgung von Menschen mit Demenz im Allgemeinkrankenhaus
Kelley et al. [16]	2019	England	Ethnografische Studie	Prozess des Einbezugs Angehöriger in die Pflegeplanung und allgemeine Krankenhaus-Demenzpflege
Moyle et al. [19]	2016	Australien	Explorativ-deskriptives Design	Rolle und Bedürfnisse von Angehörigen, deren Familienmitglied mit Demenz in einem Akutkrankenhaus versorgt wurde
Nufer und Spichiger [21]	2011	Schweiz	Qualitative Studie	Angehörige von Menschen mit Demenz erleben und beschreiben deren Aufenthalt auf einer Akutstation und ihre eigene Zusammenarbeit mit den Fachpersonen

## Material und Methode

Zur Erstellung der Literaturübersicht wurde eine systematische Literaturrecherche durchgeführt [2, 20, 22]. Hierbei erfolgte auf Basis der oben genannten Fragestellung zunächst eine Annäherung an Suchkomponenten und Schlüsselwörter unter Verwendung des PICO-Schemas (Tab. 2). Zusätzlich wurden Ein- und Ausschlusskriterien formuliert und das Rechercheprinzip definiert. Eingeschlossen wurden alle Publikationen in Deutsch und Englisch ab dem Jahr 2000, da seit dieser Zeit die besondere Situation von Patienten mit Demenz im Akutkrankenhaus zunehmend in den Fokus rückte. Vor allem in den letzten 10 Jahren gibt es vermehrt Studien zu dieser Thematik. Ausgeschlossen wurden Publikationen mit den Schwerpunkten Delir oder leichte kognitive Beeinträchtigung („mild cognitive impairment“ [MCI]), da MCI und Delir diagnostisch von der Demenz abgegrenzt sind [24, 25]. Das MCI kann eine Vorstufe von Demenz sein – muss es aber nicht. Das Delir ist ein akuter, durch Erkrankung oder Arzneimitteltoxizität verursachter Verwirrtheitszustand, der häufig reversibel ist. Weitere Ein- oder Ausschlüsse wurden vorab nicht formuliert, da das Rechercheprinzip primär sensitiv angelegt war.

**Table 2:** 

*P*opulation	Angehörige von Patienten mit Demenz
*I*ntervention	Begleitung während des Krankenhausaufenthaltes
*C*omparison	Auswirkungen bei Abwesenheit von Angehörigen
*O*utcome	Sichtweisen im Hinblick auf die Begleitung
Time	Seit 2000 (20 Jahre)
Setting	Krankenhaus, akutstationär

Im nächsten Schritt wurden zu den Suchbegriffen Suchmatrizen erstellt, um verwandte Begriffe und Synonyme auf Deutsch und Englisch zu ermitteln. Anschließend wurden die zu durchsuchenden Fachdatenbanken benannt: *Medline* (*PubMed*), *Cochrane, CINAHL* und *GeroLit*. Jede Datenbank hat ihre eigene Verschlagwortung, sodass die jeweils zutreffenden Schlagwörter identifiziert wurden. Durch Kombination von Stich- und Schlagwörtern je Suchkomponente und Fachdatenbank entwickelte sich der jeweilige Such-String. Hierbei fanden Trunkierungen und Boole’sche-Operatoren Anwendung.

**Figure 2:**
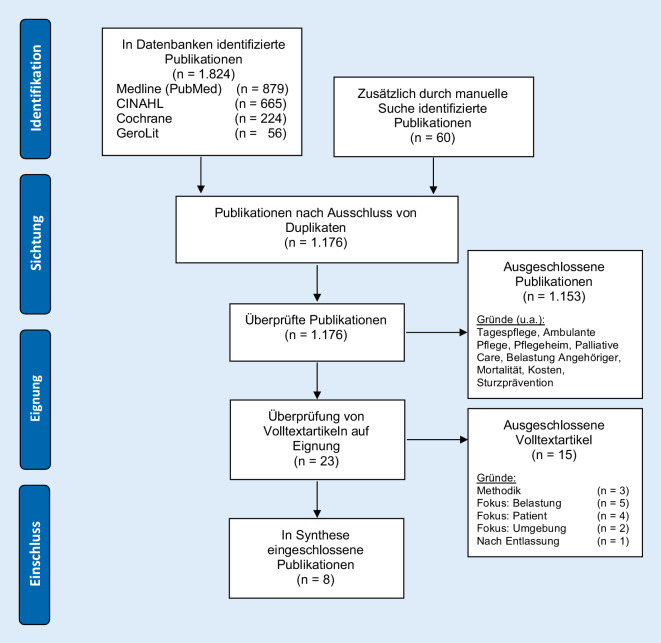

Die Datenbankrecherche erfolgte von Mai bis Juli 2020. Sie wurde durch eine manuelle Recherche in deutschsprachigen Fachzeitschriften ergänzt (*Zeitschrift für Gerontologie und Geriatrie, Pflegewissenschaft, PflegeZeitschrift, Pflege, Pflege & Gesellschaft*). Eine Übersicht zum Screeningprozess, einschließlich der Ausschlussgründe von Publikationen, zeigt das Flussdiagramm in Abb. 2. Zu 23 Volltextartikeln erfolgte eine detaillierte inhaltliche Bewertung und Analyse.

Insgesamt konnten international 5 qualitative Studien, eine Sekundäranalyse, eine Mixed-Methods-Studie und ein systematisches Review für die nähere Betrachtung und Beantwortung der Fragestellung herangezogen werden (Tab. 1).

## Ergebnisse

Alle einbezogenen Publikationen rekonstruieren das Erleben und die Sicht von Angehörigen bei einem Krankenhausaufenthalt der Person mit Demenz. Trotz der unterschiedlichen Gesundheitssysteme in den Ländern ähneln sich die Aussagen, Schilderungen und Wünsche von Angehörigen im Hinblick auf die Begleitung von Patienten mit einer Demenz im akutstationären Setting sehr. Bei der Analyse wurden 5 Themengebiete identifiziert: Gründe für die Begleitung, Art der Begleitung, Auswirkungen der Abwesenheit von Angehörigen sowie hinderliche und förderliche Faktoren für die Begleitung.

### Gründe für die Begleitung durch Angehörige

Für Angehörige existieren vielfältige Gründe, Patienten mit Demenz während des Krankenhausaufenthaltes umfassend zur Seite zu stehen. Dabei ist ihr Hauptanliegen sicherzustellen, dass die Personen mit einer demenziellen Erkrankung in bestmöglicher Art und Weise versorgt und ihre Grundbedürfnisse erfüllt werden [3, 14, 16, 21].

Unabhängig von der Art der Beziehung besteht bereits vor dem Krankenhausaufenthalt eine enge emotionale und pflegerische Verbindung zwischen dem Patienten und der primären Bezugsperson. Durch ihre Gegenwart wird dem Patienten ein Gefühl der Vertrautheit und Sicherheit vermittelt. Verstärkt wird dieses durch bekannte Routinen und das Aufrechterhalten pflegerischer Abläufe [10, 14–16, 21].

Angehörige stehen dem Fachpersonal als Berater und Experten im Umgang mit dem Menschen mit Demenz zur Verfügung. Sie wissen um deren grundlegende Bedürfnisse, Präferenzen, Verhaltensweisen und Ängste. Vor allem im fortgeschrittenen Stadium der Demenz, wenn eine Kommunikation nur noch schwer gelingt, finden sie einen Zugang zum Patienten [3, 6, 10, 14–16, 21].

In dieser Hinsicht können Angehörige das medizinische und pflegerische Fachpersonal in die Lage versetzen, mit dem Patienten zu interagieren. Hierbei geht es z. B. um das Erkennen und Interpretieren spezifischer Äußerungen wie Hunger, Durst, Schmerz oder Angst. Werden diese vom Fachpersonal richtig eingeschätzt, können potenzielle Probleme in der Versorgung des Patienten frühzeitig gelöst oder gänzlich vermieden werden [6, 19]. Allerdings erleben Angehörige sehr häufig, dass das Fachpersonal zu wenig demenzspezifisches Wissen besitzt, um adäquat reagieren zu können [3, 15, 16, 21]. In diesen Fällen treten sie als Fürsprecher für den Patienten ein, indem sie die Erfüllung seiner Bedürfnisse aktiv einfordern [3, 10, 14, 15, 19, 21]. Angehörigen ist es sehr wichtig, für den demenziell Erkrankten und in seinem Sinne zu sprechen, sofern er dazu selbst nicht mehr in der Lage ist. So sollen die Versorgung positiv beeinflusst und eine potenzielle Verschlechterung des physischen und mentalen Zustands abgewendet werden [3, 15].

Unabhängig davon, ob Angehörige sich aus Pflichtgefühl, Sorge oder Fürsorge um den Patienten kümmern – sie wollen der ihnen obliegenden Verantwortung für den Patienten gerecht werden [14].

### Art der Begleitung

Viele Angehörige sind mehrfach täglich im Akutkrankenhaus. Sie nutzen die Besuche, um dem Patienten eine Verbindung zur Außenwelt wie auch zu seinem Selbst und seiner Identität aufrechtzuerhalten. Hierzu werden Nachrichten und Neuigkeiten mitgeteilt, Fotos gezeigt oder eigene Kleidungsstücke und Gegenstände mitgebracht. Auch Spaziergänge werden unternommen [6, 16, 19]. Die soziale Interaktion soll ein Gefühl von Vertrautheit in einer unbekannten Umgebung erzeugen. Zusätzlich erfolgt die Begleitung in der Absicht einer Stimulation und des Vorbeugens oder Linderns von Langeweile und Einsamkeit [3, 6, 19].

Manche Angehörige übernehmen darüber hinaus konkrete pflegerische Handlungen: Unterstützung bei der Medikamenteneinnahme, Hilfe bei der Nahrungs- und Flüssigkeitsaufnahme, Übernahme der Körperpflege sowie Begleitung bei Toilettengängen [3, 6, 10, 14–16, 19]. Diese Tätigkeiten erfolgen entweder aus dem Wunsch, für Normalität zu sorgen, oder aus dem Bestreben, das Pflegepersonal zu entlasten. Indes gibt es auch Angehörige, die explizit nicht in die direkte Pflege involviert werden möchten [3].

Als Kenner des Patienten geben Angehörige gerne Informationen über dessen persönliche und medizinische Historie an das Fachpersonal weiter [19, 21]. In dieser Expertenrolle sind sie in der Lage, Veränderungen im Zustand des Patienten, beispielsweise hinsichtlich seiner Gesundheit, Ernährung oder des Befindens, zeitnah zu registrieren und Anpassungen einzufordern [21]. Ferner können sie dazu beitragen, schwierige Situationen, z. B. ausgelöst durch Unruhe, Angst oder aggressives Verhalten, aufzulösen [6, 10, 14, 21].

Angehörige sind sehr darum bemüht, bei Visiten anwesend zu sein. Zum einen, um Informationen zum Behandlungs- und zum Genesungszustand des Patienten zu erhalten und Fragen zu stellen. Zum anderen, um ihre patientenbezogene Erfahrung zu äußern.

### Auswirkungen der Abwesenheit von Angehörigen

Demenziell erkrankte Patienten zeigen unterschiedliche Verhaltensweisen, wenn Angehörige nicht anwesend sind. So können sie diese vermissen, suchen oder nach ihnen rufen. Auch die Ablehnung von Nahrung, das Verwehren medizinisch-pflegerischer Maßnahmen oder das Verweigern verordneter Medikamente ist möglich. Eine weitere Auswirkung kann eine unruhebedingt erhöhte Sturzgefahr sein. Zudem ist häufig eine ungenügende Schmerzlinderung durch Fehlinterpretation der Symptomäußerung zu beobachten. Schmerz äußert sich bei Patienten mit Demenz oftmals atypisch, z. B. durch Unruhe, Bewegungsverweigerung, Abwehrverhalten bis hin zu körperlicher Aggression [14, 16, 21]. Fehlinterpretationen können zu einer Negativspirale in der Interaktion zwischen den Patienten mit Demenz und dem Klinikpersonal führen [10].

Natürlich gibt es auch Pflegefachkräfte, die in diesen Fällen dem Patienten positiv begegnen und flexibel eine individuelle Lösung finden [3].

### Hinderliche Faktoren für die Begleitung

Angehörige sehen sich bei der Begleitung des Patienten mit Demenz mit unterschiedlichen Umständen konfrontiert, die sich negativ auf die Versorgung des Patienten und limitierend auf ein wirksames Handeln des Angehörigen auswirken.

Hierzu gehören starre Besuchszeiten, vorgegebene Stationsabläufe sowie fehlende Kenntnisse und Handlungsansätze im Umgang mit Menschen mit Demenz. Das Akutkrankenhaus ist nicht auf die Begleitung durch Externe ausgerichtet, da diese nicht geplanter Teil des Systems oder Prozesses sind [3, 10, 14, 16, 19]. Fehlende stationsübergreifende Standardansätze und primäre Ansprechpartner für Patienten und Angehörige führen dazu, dass Informationen zum Patienten mehrfach wiederholt werden müssen [6, 21]. Gezielter Informationsaustausch und Wissensweitergabe zwischen Angehörigen und Fachpersonen werden dadurch deutlich erschwert. Auch ist vielen Angehörigen nicht klar, was von ihnen erwartet wird: Sind sie Besucher oder aktiv pflegende Angehörige? Wird die Weitergabe ihres Wissens gewünscht oder abgelehnt? Dürfen sie sich einbringen und, wenn ja, in welchem Umfang [16, 19]?

Vielfach erfolgt eine mangelnde oder unglückliche Kommunikation, z. B. das Reden über den Patienten in seiner Anwesenheit, ohne ihn einzubeziehen [10, 16, 21]. Des Weiteren fühlen sich Angehörige regelmäßig nicht ernst genommen oder beachtet, und ihre Informationen werden ignoriert. Kurze, von Eile geprägte Gespräche unter Verwendung einer unverständlichen Fachsprache erschweren Angehörigen eine umfassende Versorgung ihres Anvertrauten. Dadurch wird ihre wichtige Rolle als Fürsprecher erschwert, und sie werden nicht in die Versorgungssituation integriert [3, 6, 14, 15, 19, 21]. Manche Angehörige erleben, dass ihre Hilfe auf der Station unerwünscht ist oder sogar verhindert wird [15].

### Förderliche Faktoren für die Begleitung

Gegenüber den soeben dargestellten, erschwerenden Bedingungen gibt es auch Faktoren, die eine Begleitung unterstützen. Wenn das Fachpersonal den Angehörigen respektvoll gegenübertritt, zuhört und ein Gefühl von Willkommen-Sein auf der Station erzeugt, wird ihnen die Begleitung erleichtert. Zudem wird dadurch die Basis für eine gelingende gegenseitige Kommunikation gelegt [6, 10, 14–16, 21]. Werden Angehörige zusätzlich in Entscheidungen zur medizinischen Behandlung und pflegerischen Versorgung einbezogen, fühlen sie sich als Teil des Teams, das sich gemeinsam um das Wohlergehen des Patienten kümmert.

Eine bewusste Beziehungsgestaltung ist essenziell für eine gute Zusammenarbeit. Wichtig ist außerdem eine feste Ansprechperson für den Angehörigen, um sich bestmöglich gegenseitig zu informieren [3, 6, 14, 15, 19, 21].

Ein weiteres unterstützendes Element ist geschultes Fachpersonal, dass sich merklich mit der besonderen Versorgungssituation älterer Patienten auskennt und die persönlichen Bedürfnisse des Patienten berücksichtigt [6, 14, 15, 19]. Wird zudem ein personenzentrierter Ansatz verfolgt, haben Angehörige das Gefühl, dass sich um den Patienten gekümmert wird und er gut versorgt ist [15, 19].

## Diskussion

Aus den dargestellten Ergebnissen lässt sich ableiten, dass Angehörige als Bindeglied und Vermittler zwischen den Welten des Patienten mit Demenz und des Krankenhauspersonals tätig sein können und dieses in der Praxis auch bereits umsetzen. Angehörige begleiten Patienten mit Demenz im Akutkrankenhaus, um ihnen ein Gefühl von Vertrautheit und Sicherheit zu vermitteln, eine gute Versorgung sicherzustellen und bestehende Routinen aufrechtzuerhalten.

Für die Mitarbeiter im Akutkrankenhaus stehen Angehörige als Experten und Berater im Umgang mit den demenziell Erkrankten zur Verfügung. In dieser Rolle unterstützen sie die Interaktionsfähigkeit des Personals mit den Patienten, einschließlich des Lösens schwieriger Situationen. Andererseits treten Angehörige gegenüber den Mitarbeitern im Bedarfsfall als Fürsprecher der Patienten auf. Mit diesen Tätigkeiten reduzieren sie ein Interaktionsdefizit zwischen den Patienten und Fachkräften und vergrößern den Interaktionsbereich.

Die Übernahme pflegerischer Handlungen bedient gleichzeitig beide Welten. In Richtung Patient soll ein Stück Normalität vermittelt werden; in Richtung Personal geht es um dessen Entlastung.

Bei der Vermittlungstätigkeit lassen sich Faktoren benennen, die diese erschweren: z. B. mangelnde Kommunikation seitens des Personals, fehlender Einbezug der Angehörigen, starre Abläufe oder fehlende demenzspezifische Kenntnisse des Personals. Wenn Angehörige sich hingegen auf der Station willkommen fühlen, einen festen Ansprechpartner haben, auf geschultes Personal treffen, einbezogen und integriert werden, können sie als Vermittler und verbindendes Element leichter tätig sein.

Einzelne Kliniken haben bereits die Integration von Angehörigen demenzkranker Patienten in ihr Versorgungskonzept aufgenommen [29]. Die regelmäßige Empfehlung, Angehörige und Patienten mit Demenz als Dyade anzusehen, unterstreicht die Relevanz dieser Thematik [5, 15, 18, 26].

Auch wenn Angehörige als Bindeglied vermittelnd tätig sein und ein Interaktionsdefizit reduzieren können, wollen nicht alle diese Funktion ausüben [3, 14, 21]. Darüber hinaus kann die Begleitung für Patienten, die Gewalt oder Misshandlungen durch Angehörige erleben, kontraproduktiv sein [12].

## Limitationen und weiterer Forschungsbedarf

- Bei der Literaturrecherche wurde die Suche auf Publikationen in Deutsch und Englisch begrenzt. Dies schließt anderssprachige relevante Literatur aus.
- Keine der vorliegenden Publikationen behandelt die Situation in Deutschland.
- Die einbezogenen Veröffentlichungen sind aufgrund anderweitiger Fragestellungen entstanden. Die hier dargestellten Ergebnisse und die daraus resultierende Diskussion sind thematische Ableitungen aus diesen Publikationen.
- Eine Überprüfung der Ableitungen und die Untersuchung möglicher Limitationen bei der Begleitung demenzkranker Patienten durch Angehörige im Akutkrankenhaus sollten durch weitere Studien erfolgen.

## Fazit für die Praxis

- Angehörige können v. a. dann zwischen Patienten mit Demenz und medizinisch-pflegerischem Personal im Akutkrankenhaus als Bindeglied tätig sein, wenn dies vonseiten des Personals und der Organisation gewünscht und unterstützt wird. Hilfreich sind z. B. eine offene, zugehende und wertschätzende Haltung, Kommunikation und Interaktion sowie eine zentral zuständige Ansprechperson.
- Das Fachpersonal sollte Angehörige als Experten und Berater im Umgang mit Patienten mit Demenz anfragen und einbeziehen.
- Angehörige und Patienten mit Demenz haben eine dyadische Beziehung. Angehörige fühlen sich für die Patienten und ihr Wohlergehen verantwortlich und können diesbezüglich als Fürsprecher gegenüber den Krankenhausmitarbeitern auftreten.
